# Prebiotic Effect of Lycopene and Dark Chocolate on Gut Microbiome with Systemic Changes in Liver Metabolism, Skeletal Muscles and Skin in Moderately Obese Persons

**DOI:** 10.1155/2019/4625279

**Published:** 2019-06-02

**Authors:** Maria Wiese, Yuriy Bashmakov, Natalia Chalyk, Dennis Sandris Nielsen, Łukasz Krych, Witold Kot, Victor Klochkov, Dmitry Pristensky, Tatyana Bandaletova, Marina Chernyshova, Nigel Kyle, Ivan Petyaev

**Affiliations:** ^1^Department of Food Science, University of Copenhagen, Rolighedsvej 26, 1958, Frederiksberg, Denmark; ^2^Lycotec Ltd., Granta Park, Cambridge, CB21 6GP, UK; ^3^State Medical University, Research Institute of Cardiology, 12 Chenyshevskogo Str, 410028 Saratov, Russia; ^4^Department of Environmental Sciences, Aarhus University, Denmark; ^5^DiagNodus Ltd., Granta Park, Cambridge, CB21 6GP, UK

## Abstract

Lycopene rich food and dark chocolate are among the best-documented products with a broad health benefit. This study explored the systemic effect of lycopene and dark chocolate (DC) on gut microbiota, blood, liver metabolism, skeletal muscle tissue oxygenation and skin. 30 volunteers were recruited for this trial, 15 women and 15 men with a mean age of 55 ± 5.7 years and with moderate obesity, 30 < BMI < 35 kg/m^2^. They were randomized and divided into five equal interventional groups: three received different formulations of lycopene, one of them with a 7 mg daily dose and two with 30 mg; another group was given 10 g of DC with 7 mg lycopene embedded into its matrix, and the last group received 10 g DC. The trial was double-blinded for the three lycopene groups and separately for the 2 DC groups; the trial lasted for 1 month. By the end of the trial there were dose-dependent changes in the gut microbiota profile in all three lycopene groups with an increase of relative abundance of, e.g.,* Bifidobacterium adolescentis* and* Bifidobacterium longum*. This was also accompanied by dose-dependent changes in the blood, liver metabolism, skeletal muscle and skin parameters. Consumption of DC resulted in increased relative abundance of, e.g.,* Lactobacillus *and a reduction of corneocyte exfoliation. This is the first study which reports the prebiotic potential of lycopene and DC.

## 1. Introduction

Carotenoids are essential micronutrients, which cannot be synthesized by humans and must be obtained from food. Lycopene, the red pigment of tomatoes, watermelon, and some other fruits, is one major carotenoids. Intake of lycopene rich food has been linked to lower prevalence of cardiovascular disease, stroke [1] and some forms of cancer [2, 3]. Limited interventional clinical studies have indicated its therapeutic ability to slow down development of carotid atherosclerosis [4], anti-infective and anti-inflammatory properties [5], improvement of parameters associated with prostate hyperplasia [6], benefit in management of prostate cancer [7] and help to protect skin from UV damage [8, 9].

The concentration of lycopene in blood and body tissues is highly variable and depends on dietary habits and age and has also been related to health status. For example, the plasma or serum concentration could vary from about 60 ng/ml, or below, to 600 ng/ml or above [10]. A reduced level in the body could be due to three major reasons: either low dietary intake, impaired lycopene absorption and processing for example in older persons or those with metabolic syndrome, which results in poor bioavailability of this carotenoid or due to its accelerated depletion as a result of ongoing free radical pathologies in the body.

The current consensus on the broad beneficial effects of lycopene on health exists with regard to its powerful antioxidant properties and the related protection of lipoproteins and other lipid structures from oxidative damage, which typically are associated with a number of pathological conditions [1, 11].

It has been demonstrated that cocoa flavanols have a systemic effect in healthy volunteers with prebiotic activity on the gut microbiome and reduction of blood lipoproteins produced by the liver [12]. However, in real life these flavanols usually consumed by humans in a food form of chocolate, which contains cocoa butter, triglyceride lipids, other cocoa ingredients, and cocobiota metabolites [13]. Therefore, another objective of our study was to assess whether regular consumption of dark chocolate, DC, would have a similar effect as the cocoa flavanols drink. Although there were a number of reports, which demonstrated a positive impact of DC on blood lipids [14, 15], to the best of our knowledge this is the first study investigating the effect of lycopene and DC on the gut microbiota in middle-aged subjects with moderate obesity, 30 < Body Mass Index, BMI < 35 kg/m^2^. For this purpose we used GA lycopene, which is specifically formulated to overcome reduced bioavailability of this carotenoid in older people and in individuals with metabolic syndrome (Lycotec, UK). We found that continuous administration of this product for 4 weeks resulted in significant prebiotic effects. These positive changes in the gut microbiota profile were accompanied by systemic improvement of different physiological parameters of the participants from blood and liver metabolism to peripheral tissues including skin.

## 2. Methods

*Study Design*. In total 30 volunteers were recruited to take part in the study, 15 male and 15 female, all Caucasian within the age span of 40–68 and median 55 ± 5.7 years. They were randomized and divided into five groups of equal size. Group I received a daily dose of 10 g dark chocolate with 7 mg lycopene by a proprietary protocol guaranteeing its maximum embedment into the lipid part of the chocolate, L-Tug, and on another optimal lycopene coating of chocolate crystals and formation of coco- lycosomes, DCL [16]. Group II received daily one capsule of 7 mg GA lycopene formulated with medium saturated fatty acids, GAL-MSFA, Group III one capsule daily of 30 mg GAL-MSFA, group IV one capsule daily 30 mg of GA lycopene formulated with polyunsaturated fatty acids, GAL-PUFA, and group V 10 g of the control dark chocolate daily. Three GAL groups received blinded lycopene capsules, as two other groups received blinded DC products. 

*Products*. All products for the trial were developed and made by Lycotec Ltd. (Cambridge, United Kingdom). The product was especially designed to improve lycopene bioavailability in middle-aged persons, 50 years old or above, or in those who have such conditions as metabolic syndrome, fatty liver, etc. [17]. It contained phosphatidylcholine, which serves as a principle scaffolding element for incorporation of lycopene during lipoprotein intracellular reassembly, the process that is essential for lycopene transportation but impaired in the above individuals.

There were two formulations of GAL, for two different nutraceutical applications, which were applied in this study. The first was with a blend of MSFA to facilitate formation of small-medium chylomicrons, which would be transported by the portal vein for liver targeting delivery of lycopene. The second one was a blend with PUFA to facilitate formation of larger chylomicrons, which would be transported by the thoracic duct for the systemic blood circulation bypassing the liver. All GAL products were made in gelatin capsule.

For the control DC and DCL Green & Black's 70% dark chocolate was used. It was made from Trinitario cocoa beans and contained 42% fat, of which saturates were 25%; carbohydrates 36.5%, of which sugars were 28.5%; fibre 10%, protein 9.1%, salt 0.13%. Each 10 g bar contained 1.5 mg of catechins, 6.6 mg of epicatechins, 1.9 mg of dimer-B2, 7.5 mg of caffeine, 75 mg of theobromine, 75 *μ*g of phenylethylamine, 55 *μ*g of serotonin, and ≤ 0.1 *μ*g of resveratrol.

Both capsule and chocolate products were advised to be taken once a day after the main meal.

The duration of the trial was 1 month.

The treatment part of the study and the blood analysis were conducted at the Institute of Cardiology, the Ministry of Health of the Russian Federation (Saratov, Russian Federation) by Lycotec Ltd. (Cambridge, United Kingdom). The protocol was approved by the Local Ethics Committee (FGBU SarNIIK18.02.2014). Trial registration number was ACTRN12618000715279. All patients were informed of the purpose and goals of the study and had signed a consent form before enrolment and participation in the study.

The stool microbiota analysis was made by the Department of Food Science in the Section of Food Microbiology, at the University of Copenhagen in Denmark.

The skin samples were analysed by Lycotec in Cambridge.

### 2.1. Inclusion/Exclusion Criteria

*Inclusion Criteria were as follows*:

- ability to sign an informed consent,
- nonsmokers or light-to-moderate smokers (≤10 cigarettes daily),
- moderately obese with BMI between 30 and 35 kg/m^2^,
- with elevated serum markers of inflammatory oxidative damage, IOD ≥ 40* μ*M/mL and oxidative stress, LDL-Px, ELISA × 10^3^ ≥ 200,
- no participation in other dietary trials during the last 3 months before enrolment and duration of study,
- willingness and ability to comply with the study protocol for the duration of the study.

*Exclusion criteria* were as follows:

- unwillingness to sign informed consent,
- unable to comply with the protocol for the duration of the study,
- history of myocardial infarction in the 3 months preceding the study, ejection fraction (EF) < 45%,
- significant medical condition that would impact safety considerations (e.g., significantly elevated liver enzymes, hepatitis, severe dermatitis, uncontrolled diabetes, cancer, severe GI disease, fibromyalgia, renal failure, recent CVA (cerebrovascular accident), pancreatitis, respiratory diseases, epilepsy, etc.),
- compulsive alcohol abuse (>10 drinks weekly),
- or regular exposure to other substances of abuse,
- participation in other nutritional or pharmaceutical studies,
- resting heart rate of >100 beats per minute or <50 beats per minute,
- positive test for tuberculosis, HIV, or hepatitis B,
- inability to tolerate phlebotomy,
- special diets in the 4 weeks prior to the study (e.g., liquid, protein, and raw food diet),
- tomato or DC intolerance.

### 2.2. BMI, Pulse Rate, and Systolic and Diastolic Blood Pressure

Measurements of body mass index, BMI, body mass of the patients and their height were carried out in the morning and BMI was calculated in kg/m^2^. Pulse rate, systolic and diastolic blood pressure, SBP, and DBP were recorded three times on the left arm of the seated patient after 15 min of rest. The time between measurements was greater than 2 minutes. The mean result for each parameter was calculated. All body and vascular parameters were recorded in the morning between 8 and 10 am.

*Tissue Oxygenation*. Thenar eminence and forearm muscles of the patients were used as a tissue target for the assessment of oxygen saturation, StO_2_, or combined level of oxygenated haemoglobin and myoglobin. StO_2_ was assessed by continuous wavelength near-infrared spectroscopy, NIRS, with wide-gap second-derivative (In Spectra, Hutchinson Technology, MN, USA). The measurements were taken at different time points. The recording was initiated after 15 min of rest in a supine position before occlusion of the brachial artery. It was then continued during stagnant ischemia induced by rapidly inflating the cuff to 50 mm Hg above systolic BP. The ischemia lasted for 3 min, and the recording period lasted for another 5 min after that until StO_2_ was stabilized. The area under the hyperaemic curve, AUC, of the recorded signal for the settling time in the postocclusion period was then calculated as described earlier in % O_2_/minute [17, 18].

*Samples Collection*. Blood was collected by phlebotomy in the morning, in the hospital, and from the arm veins of patients following night fast. The serum was separated from the rest of the clotted mass by centrifugation; aliquots were then stored in code-labeled tubes for blinded analysis and stored at −80°C until use.

For sample collection from the surface of the facial skin and samples of the cerumen all study participants were requested to avoid facial and ear hygienic manipulations for 24 hours before sampling, which was carried out in the morning in parallel with blood sample collection. Skin surface sample collection and preparation were performed as previously described [19]. Briefly, samples were collected using polyester swabs from the surface of the facial skin (the sides of the nose). During the procedure two samples were taken (one swab per side). Each collected sample was placed on the surface of a microscope slide. A second microscope slide was pressed against the surface of the first one. This procedure provided a pair of identical smears. All slides with collected samples were coded to provide sample anonymity for blinded analysis and stored at −20°C until further analysis.

The stool samples were collected either on the morning or night before the day of the visit to the hospital. Participants did this collection themselves, at the convenience of their home, in the morning on the day of visiting clinic. A special kit and sample containers were provided by the trial team. The collected samples were labeled and stored at −80°C until analysis.

### 2.3. Gut Microbiome Analysis

#### 2.3.1. DNA Extraction

Genomic DNA was extracted from 200 mg stomached fecal material (stomacher 2x 60 sec at mid speed) using the Power Soil Kit protocol (MoBio Laboratories). The FastPrep bead-beating step was performed in 3 cycles of 15 s each at a speed of 6.5 M/s in a FastPrep-24TM Homogenizer (MP). DNA quantity and quality were measured using a NanoDrop 1000 (Thermo Scientific), 16S rRNA gene library preparation. The fecal microbiota composition was determined using tag-encoded 16S rRNA gene MiSeq-based (Illumina, CA, USA) high throughput sequencing. In brief the V3 region of the 16S rRNA gene was amplified using primers compatible with the Nextera Index Kit (Illumina) NXt_338_F:5′- TCGTCGGCAGCGTCAGATGTGTATAAGAGACAGACWCCTACGGGWGGCAGCAG -3′ and NXt_518_R: 5′- GTCTCGTGGGCTCGGAGATGTGTATAAGAGACAGATTACCGCGGCTGCTGG -3^′..^ [20]; the PCR reactions and library preparation were conducted as described in [21].

#### 2.3.2. High throughput Sequencing and Data Treatment

The raw dataset containing pair-ended reads with corresponding quality scores were merged and trimmed using fastq_mergepairs and fastq_filter scripts implemented in the UPARSE pipeline. The minimum overlap length was set to 10 base pairs (bp). The minimum length of merged reads was 150 bp, the maximum expected error E was 2.0, and the first truncating position with a quality score was N≤4. Purging the dataset from chimeric reads and constructing de novo Operational Taxonomic Units (OTU) was conducted using the UPARSE pipeline [22]. The Green Genes (13.8) 16S rRNA gene collection was used as a reference database [23]. Quantitative Insight Into Microbial Ecology (QIIME) open source software [24] (1.7.0 and 1.8.0) was used for the subsequent analysis steps. Principal coordinate analysis (PCoA) plots were generated with the Jackknifed Beta Diversity workflow based on 10 UniFrac distance metrics calculated using 10 subsampled OTU tables. The number of sequences taken for each jackknife subset was set to 90% of the sequence number within the most indigent sample, hence 10000 reads per sample. Permutational Multivariate Analysis of Variance (PERMANOVA) was used to evaluate group differences using weighted, unweighted, and generalized UniFrac distance metrics that were generated based on rarefied (10000 reads/sample) OTU tables. The relative distribution of the genera registered was calculated for unified and summarized in genus level OTU tables. Alpha diversity measures expressed as observed species values (sequence similarity 97%) were computed for rarefied OTU tables (10000 reads/sample) using the alpha rarefaction workflow. Differences in alpha diversity were determined using a t-test-based approach employing the nonparametric (Monte Carlo) method (999 permutations) implemented in the comparative alpha diversity workflow. The ANOVA determined significance of quantitative (relative abundance) association of OTUs with given categories; p values were False Discovery Rate (FDR) corrected. These were calculated based on 1000 subsampled OTU tables rarefied to an equal number of reads (10000 reads/sample). Spearman correlations between the taxa relative abundance (at the OTU level and summarized to the species level) and the host parameters were conducted using observation metadata correlations script (Qiime 1.9.1). Fisher z-transformation method with 1000 permutations was used for calculating p values. The influence of explanatory variables (host parameters) and OTUs relative abundance (species level) was tested with the rda function (vegan R-package), using the ANOVA-like permutation tests (1,000) to determine the significance of the putative constrains effects [25].

*Biochemistry*. Glucose, total cholesterol, triglycerides, high density cholesterol, low density cholesterol and C-reactive protein were determined using commercially available analytical kits according to the manufacturers' instructions (ByoSystems, R&D Systems).

*Lycopene Quantitative Analysis*. The lycopene concentration in all serum samples was measured in duplicate by high-performance liquid chromatography [26] with modifications. Briefly, 400 *μ*l of serum was mixed with 400 *μ*l of ethanol and was extracted twice with 2 ml hexane. The combined hexane layers were evaporated to dryness in a vacuum (Scan Speed 32 centrifuge) and the residue reconstituted to a volume of 100 *μ*l in sample solution (absolute ethanol – methylene chloride, 5:1, v/v). The specimens were centrifuged again (15 minutes at 10,000 g) and clear supernatant was transferred to HPLC vials. Five microliters of the extract was injected into an Acquity HSS T3 75x 2.1mm 1.8 *μ*m column (Waters, USA) preceded by a Acquity HSS T3 1.8 *μ*m VanGuard precolumn (Waters, USA) and eluted isocratically at 45°С with the mobile phase (acetonitrile – 0.08% phosphoric acid solution - tert-Butyl methyl ether, 70:5:25, v/v/v) at a flow rate of 0.5 ml/min. The lycopene peak was detected by a Photodiode Array Detector (Waters, USA) at 474 nm. The peak area was measured using Empower 3 software (Waters, MA). The lycopene concentration in serum samples was calculated by reference to an analytical standard (lycopene from tomato, L9879, Sigma, USA).

*Inflammatory Oxidative Damage (IOD).* Serum samples were incubated in 0.05 M PBS acetate buffer (pH 5.6) overnight, to imitate the type of oxidative damage which occurs during the release of lysosomes following neutrophil degranulation. The following morning the reaction was stopped using trichloroacetic acid. The concentration of the end products such as malondialdehyde (MDA), and other possible thiobarbituric acid reactive substances (TBARS), was then measured by colorimetry [27] using reagents and kits from Cayman Chemical (MC, USA).

*LDL-Px and Lipoprotein O*_2_. Activity of serum LDL peroxidase proteins, which include IgG with superoxide dismutase activity, was measured as described previously [28]. Plasma oxygen, which carried by blood lipids/lipoproteins, was measured by catalymetry [29].

*Statistics*. For the assessment of normally distributed parameters the Shapiro-Wilk method was used. Student's* t*-test was then applied for both paired and unpaired samples. In cases where parameters were not normally distributed the Mann–Whitney test and Kruskal-Wallis test were used. ANOVA and ANCOVA were used with post hoc analysis (Statistica 9 suite, StatSoft, Inc.). Statistical significance between two-tailed parameters was considered to be P<0.05.

## 3. Results

Baseline characteristics of the participants are presented in Table 1 and were comparable between all five groups.

### 3.1. Blood and Liver Metabolism

Ingestion of lycopene products for one month, either in the capsule format or in the chocolate matrix, resulted in a significant increase of its concentration both in the serum and in the ear skin excretion (Table 2).

Supplementation with GAL-MSFA resulted in a dose-dependent significant reduction of markers of oxidative damage and inflammation. 7 mg of lycopene was able to reduce IOD and LDL-Px, by the end of the month, by 49 *μ*M MDA, or by 35%, and by 200 ELISA units, or by 36%, while 30 mg reduced these parameters by 69, or by 60%, and 285, by 43%, accordingly. 30 mg of GAL-MSFA was 3 fold more effective in inhibiting IOD than the same dose of lycopene but in the GAL-PUFA formulation. This may potentially indicate on the liver origin of this blood marker. Effect of two formulations of lycopene on LDL-Px was similar (Table 2).

DC with or without lycopene had a similar effect on the inhibition of IOD as 7 mg of lycopene. Although both chocolate products were able to reduce LDL-Px, their effectiveness was below that of lycopene itself.

Administration of either formulation of GAL, or lycopene with DC complex, resulted in significant changes in the profile of fasting lipoproteins, which are assembled and produced by the liver. GAL-MSFA reduced in a dose-dependent manner both LDL concentration and triglycerides. This liver targeting formulation of lycopene, in 30 mg dose, was able to reduce the first parameter by 17 mg/dL and the second by 18 mg/dL. Supplementation with GAL-PUFA resulted in LDL reduction by 13 mg/dL and triglycerides by only 3 mg/dL. Lycopene in the L-Tug complex with dark chocolate was also able to reduce LDL; however, changes caused by the ingestion of the control DC were not significant (Table 2).

By the end of the trial there were no changes in the serum concentration of HDL, glucose and liver enzymes, ALT and AST (results are not presented).

There were noticeable improvements in the molecular oxygen metabolism in all groups. In groups supplemented with GAL-MSFA O_2_ concentration and its transportation by blood lipoproteins was significantly increased by 18-19%, p < 0.05. In the group that received GAL-PUFA this increase was lower, by 12%, p > 0.05. In the group, which received control DC, the increase in the lipoprotein O_2_ was the highest, by 44%, p <0.01.

These changes in the plasma oxygen transportation translated to benefit for peripheral tissue oxygenation but not in the control DC group. Ingestion of all lycopene products also resulted in a significant boost of tissue oxygenation in skeletal muscles. Administration of GAL-MSFA demonstrated a dose-dependent effect in changes of this parameter. However, 30 mg of lycopene in GAL-PUFA formulation was 25%, p <0.05, more effective than the same dose of lycopene but in the GAL-MSFA formulation (Table 2).

### 3.2. Skin Parameters

Supplementation of the participants with all formulations of lycopene for one month resulted in significant reversal of age-associated parameters of sebum and corneocytes. Also for the GAL-PUFA a reduction was observed, though statistically not significant. The GAL-PUFA formulation was more effective in improving cellular parameters of the skin, while GAL-MSFA was more effective for sebum parameters.

However, different to the blood parameters, observed changes in the skin, apart from those related to the sebum, did not have dose-dependency. This may indicate that even the dose of 7 mg of daily supplementation with lycopene was sufficient to reach its saturated level in this tissue by the end of the trial.

**Figure 1:**
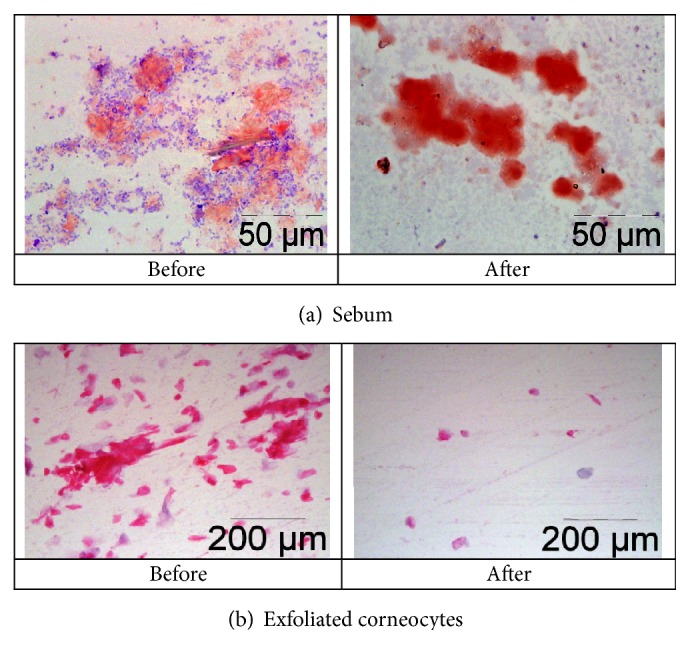
Changes in skin parameters after supplementation with 7 mg of GA lycopene for one month. Typical skin smear samples: lipid droplets of the sebum were stained with Oil Red O, and corneocytes with hematoxylin, eosin at 1,000× magnification.

The viscosity of the sebum, in terms of the size of the lipid droplets collected from the surface of the skin, was increased on average by 390 nm during this trial after supplementation by all formulations of lycopene. However, GAL-PUFA only slightly increased the diameter of the droplets, by 50 nm, while GAL-MSFA was much more effective and did it in a dose-dependent manner, by 180 nm for 7 mg of lycopene and by 480 nm for 30 mg (Table 3 and Figure 1 top images).

The rate of corneocyte exfoliation was reduced by about 23% for the former formulation and by 9-11% for the latter. Moreover, not just the rate of exfoliation was reduced by lycopene supplementation but also the damage of these cells too. The number of the clusters of cross-linked corneocytes was reduced by 36% for GAL-PUFA and by 29 to 47% by GAL-MSFA (Table 3 and Figure 1, images in the middle).

It was interesting to observe that these improvements of the sebum (Figure 1(a)) and corneocyte (Figure 1(b)) parameters were accompanied by significant reduction of the total load of the gram-negative bacteria on the surface of the skin, but only by supplementation with GAL-MSFA. In the group of GAL-PUFA there was a similar trend, but it was statistically insignificant.

In the control DC group the sebum and corneocytes parameters by the end of the trial were not affected.

### 3.3. Gut Microbiome

**Figure 2:**
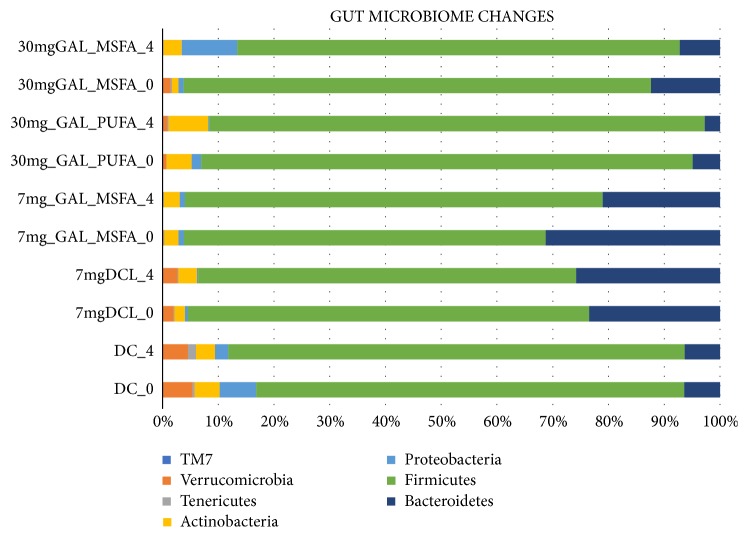
Bacterial relative abundance (Phylum level) of the different intervention groups before and after the 4 weeks of intervention as determined by 16S rRNA gene amplicon sequencing. (Group I) fortified with 7 mg lycopene - 10 g dark chocolate 7 mg DCL, (Group II) 7 mg of lycopene in a capsule 7 mg GAL-MSFA, (Group III) 30 mg of lycopene in cardiovascular GAL-MSFA, (Group IV) 30 mg lycopene liver targeting/liver health GAL-PUFA. (Group V) 10 g of dark chocolate DC.

After 4 weeks of supplementation with GA lycopene, a shift in the gut microbial communities was detected in the stool of the participants. The relative abundance of OTUs on Phyla level changed to increased relative abundance in Actinobacteria in all intervention groups, Group IV (30mg lycopene liver targeting) 4.5%- 7.12%, Group II (7mg lycopene capsule) 2.52%- 2.85%, and a significant increase was detected for Group III (30 mg lycopene cardiovascular targeting) with 1.12%- 3.22% p=0.04 (FDR corrected)(Figure 2). The separation between the week zero and week 4 of the intervention Group III is also shown in Figure 2; PERMANOVA analysis indicated a separation R= 0.250, p=0.04)

An increased dose of lycopene 30 mg Group III and IV versus Group II, 7 mg, was also reflected in an increased relative abundance of Actinobacteria (+2.6 Group IV, Group III +2.1%, Group II +0.33%). Bacteroidetes decreased in the relative abundance in all groups even though not being statistically significant (Group IV 4.92%-2.72%, Group III 12.4% to 7.2%, Group II 31.3% to 21.1%), p >0.5 (FDR corrected). No significant changes were detected for the remaining GM composition on Phyla level.

Relative abundances on the OTU species level at week 0 and week 4 for the GAL intervention groups (Group II, III, IV) with regard to formulations and dose effect are shown in Table 4.

It is evident that three different OTUs (species level cut-off), namely, OTUs representing Bifidobacterium species increased in relative abundance; these were Bifidobacterium longum, B. adolescentis, and an assigned species.

Whereas several OTUs belonging to the Prevotella genera had decreased in relative abundance over the course of the intervention, the statistically insignificant decrease in Bacteroidetes by GAL formulations seems hence to be genera specific (Table 4).

Looking at Groups I, II, and V the DC and DC-GAL (7 mg) and GAL-MSFA (7 mg) on the Phyla level we have detected a decreased relative abundance of Actinobacteria 4.4-3.4%, though not statistically significant (p>0.9) for the DC intervention; no significant changes were detected in the relative abundance of Bacteroidetes 6.4%-6.3%, Proteobacteria 6.6 -2.4% (p>0.9) for this intervention group after 4 weeks of DC intervention.

Whereas the relative abundance of Actinobacteria increased in the DC-GAL group (I) from 1.9% to 3.3% after 4 weeks of intervention, Bacteroidetes on the other hand increased slightly from 23.4 to 25.8%, Firmicutes decreased 71.7-67.8%, Proteobacteria decreased from 0.49 to 0.24%, p>0.4; these changes were not statistically significant. The relative abundance of bacteria on the species level of the DC intervention and 7 mg GAL-DC versus GAL-MSFA formulations can be seen in Table 5. For the DC intervention we detected a decrease in OTUs belonging to the Actinobacteria and an increase in Lactobacillus genera, though not statistically significant p>0.1.

**Figure 3:**
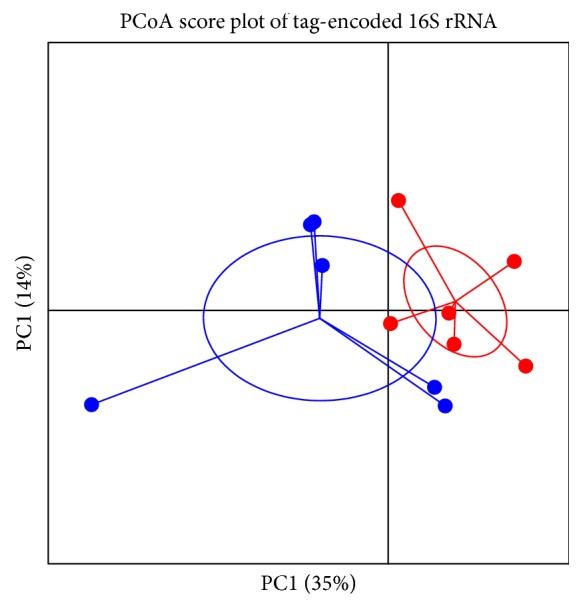
PCoA score plot of tag-encoded 16S rRNA gene amplicon sequencing data based on generalized UniFrac distance metrics (*n* = 6, for each time point) of week 0 (red dots) and week 4 (blue dots) intervention Group III. R = 0.250, p=0.04.

There were no significant correlations between the tested parameters and taxa relative abundance (raw OTU level nor summarized to the species level). No relationship between the bacterial relative abundance and host parameters could be found using the redundancy analysis (Figure 3).

## 4. Discussion

Interconnection between intestine and its flora, liver metabolism and the skin is the subject of intensive investigations [30, 31]. Therefore, in order to correct parameters of one of these organs, it is important to assess possible changes, which may develop in parallel in others. However, this alteration may not be noticeable or be minimal, and ultimately a correction will not be needed, if targeted persons are healthy and analysed parameters are within their healthy norm. Therefore, in our study we have included middle-age subjects with a mild form of obesity with blood markers of subclinical inflammation and oxidative damage.

Development of metabolic syndrome, age associated skeletal muscle loss and frailty are accompanied by ongoing, often at a subclinical level, processes of inflammatory and oxidative damage, which may lead to changes in liver metabolism, vascular functions, increased body mass and development of subclinical systemic tissue hypoxia [32, 33]. In our study we observed that supplementation with lycopene, especially formulated for effective bioavailability, in middle-aged people, had antioxidant, anti-inflammatory, and blood lipid-lowering effects, which are in accordance with earlier reports [34]. These changes in the blood markers were accompanied by or maybe resulting in the improvement of the peripheral tissue StO_2_. The main contributor into this parameter is the skeletal muscle respiration, although skin oxygenation is part of it too.

It was observed that supplementation with lycopene can increase its level in the skin tissue, which results in improving its protection from UV damage [9]. However, the fact that lycopene can be secreted to the surface of the human body either with the cerumen, which we are reporting here, or sebum (results not shown), to the best of our knowledge, has not been reported earlier.

Sebum is not only essential for skin lubrication, which prevents it from dehydration, but is also an important part of its immune system and its antibacterial acid mantle.

The sebum is also supplying antioxidants and perhaps other beneficial molecules to the surface of the skin [35]. It has been reported that with ageing the quality of the sebum is impaired, and in particular its viscosity, is increased, which is accompanied by accelerated corneocyte desquamation [19]. In our study we observed that supplementation of the diet with lycopene of middle-aged persons resulted in the restoration of the sebum viscosity, reduction of the corneocyte damage, and desquamation.

Continuous ingestion of DC had also a significant positive effect on liver associated markers of IOD and LDL-Px and also on the concentration of lipoprotein transported O_2_. However, these positive changes were not translated in improvement of skeletal muscle respiration and analysed skin parameters.

The absence of any direct correlations between relative abundance of gut taxa and analysed parameters of the blood, skeletal muscle and skin indicates a complex intertwined relationship between gut microbiome environment and the host metabolic pathways.

Prebiotics are traditionally considered to be non-digestable food ingredients, which can reach the intestine and be selectively utilized by host microorganisms conferring a health benefit [36, 37].

There are a number of molecules within food, which are not fully digestible; hence, they can reach the colon and its microbiota. Carotenoids and lycopene in particular belong to these types of partially digestible molecules [38, 39].

In our study we observed that regular intake for one month middle-aged mildly obese persons of lycopene, either in the GA formulations or in L-Tug chocolate resulted in significant changes in the profile of the gut microbiota.

GAL formulations led to a dose-dependent increase of members of the Phyla Actinobacteria, mainly driven by an increase in the relative abundance of* Bifidobacterium *spp. such as* B. longum *and* B. adolescentis*, indicating a prebiotic potential of these formulations. Further, in the DC intervention we observed an increase in a* Lactobacillus* related OTUs indicating a selective prebiotic potential of DC.

Bifidobacteria are one of the best studied genera of beneficial bacteria and often marketed as probiotics, presumably conferring a broad range of health benefits not only in the gut environment but in the whole body. This involves their ability to control bacterial and viral pathogens, stimulate local intestinal and systemic immune system, and improve lipid metabolism and weight management [40, 41]. The loss or reduction of the bifidobacterial gut associated population could be a significant factor associated with ageing-associated frailty development [42].

There is emerging evidence that dysbiosis of the gut microbiome and alteration of the associated bacterial gene pool and metabolic pathways may contribute to the development of pathogenesis of obesity [43–45] although it is still under debate whether the relative abundance of* Bacteroides* within the microbiome has been associated with obesity [46, 47].

In our study we observed that continuous intervention with GAL, DC, and DCL resulted in significant decrease in the abundance of Bacteroidetes. This could be explained either by direct action of this carotenoid, or its indirect activity via stimulation of some species of* Bifidobacteria*, or a combination of both factors.

It was interesting that observed lycopene effects on the gut bacteria, blood markers of inflammation and oxidation, lipids produced by the liver and by the skin (sebum) and peripheral tissue oxygenation were all dose-dependent.

This indicates that the observed complex of positive systemic changes could not only be a result of direct action of lycopene but also a conseqence of its indirect activity via stimulation of production of signaling metabolites* Bifidobacteria adolescentis* population with the blood, liver, skeletal; muscles and skin, which lycopene can control.

Continuous ingestion of the DC resulted in an increase in the abundance of* Lactobacillus *spp., which also constitutes a genus where several strains have been investigated and also marketed as probiotics. Increased relative abundance was also accompanied by reduction of liver associated blood markers of oxidative damage and inflammation. However, these positive changes did not positively affect skin parameters in this study group.

These results raised a number of unanswered questions, one of them being whether the observed systemic effects are specific to the lycopene molecule or other carotenoids would have similar properties.

The other question, to which we do not have an answer, is whether lycopene or dark chocolate molecules directly affected growth of the* Bifidobacteria adolescentis, B. longum,* and* Lactobacillus *or if it was their indirect effect via systemic changes. The improvement of metabolism and physiology of the gut tissues may lead to its better control of the microbiota and boost the growth of bacteria with proposed health benefits.

Whatever the nature of the prebiotic effect of lycopene, this, to the best of our knowledge, is the first report that ingestion of a carotenoid may have this new property. It is also for the first time our study demonstrated that dark chocolate has a similar effect albeit selective for a different putatively beneficial bacteria.

To conclude, the observed systemic effect of lycopene supplementation, or dark chocolate ingestion, which includes improvement of gut, blood, liver lipid metabolism and, for the former, skeletal muscles and skin parameters could be not just due to the carotenoid and dark chocolate properties themselves, but are likely also to modulate the gut microbiome increasing the relative abundance of putatively beneficial bifidobacteria and lactobacilli.

## Figures and Tables

**Table 1:** Baseline values.

BASELINE CHARACTERISTICS OF THE ENROLLED VOLUNTEERS (Mean +/- SD)					
	Groups				
	I	II	III	IV	V
Number of Patients	6	6	6	6	6

Males	3	2	4	3	3

Females	3	4	2	3	3

Age	61.8±5.9	56.2±5.9	56.1±5.8	52.1±5.1	63.2±6.1

Light/Moderate Smokers	1	1	1	1	1

Body Mass Index in kg/m^2^	32.1±2.4	32.7±3.3	33.8±3.5	31.1±3.2	31.8±2.9

Fasting Glucose mmol/dL	6.1±0.42	6.0±0.45	5.7±0.49	5.4±0.43	5.5±0.56

Total Cholesterol mg/dL	185 ± 14.3	181 ± 15.2	175 ± 14.7	187 ± 16.2	180 ± 13.9

Triglycerides mg/dl	135±14.9	136±13.8	136±13.8	127±13.1	122±13.5

LDL mg/dL	144±11.8	143±12.7	121±12.2	137±13.6	131±12.1

HDL mg/dL	41.9±3.2	46.5±4.4	51.2±4.7	49.8±4.4	44.0±4.4

Pulse rate per min	66.7±4.2	67.7±3.5	65.2±3.4	70.5±3.9	66.6±5.1

Blood Pressure					

Systolic	112±5.5	123±7.4	117±6.9	124±8.5	118±6.7

Diastolic	77.6±4.4	78.7±5.0	77.6±4.4	76.7±4.6	79±5.6

**Table 2:** Changes in blood and tissue parameters after supplementation with GA lycopene for one month.

Parameters before and after 4 weeks of the trial	Groups				
	I	II	III	IV	V
Lycopene in serum, in ng/ml					
before	110 ± 17	110 ± 12	210 ± 19	90 ± 8.4	120 ± 22
after	500 ± 52*∗∗*	310 ± 30*∗∗*	430 ± 30*∗∗*	190 ± 14*∗*	170 ± 27

Lycopene in cerumen, in ng/g					
before	53 ± 9.5	40 ± 5.5	70 ± 10.2	750 ± 93	14 ± 7.6
after	102 ± 12.4*∗*	100 ± 12.5*∗*	90 ± 11.5	2,500 ± 237*∗∗*	12 ± 5.5

Triglycerides mg/dL					
before	135±14.9	155 ± 12.1	128 ± 9.7	126 ± 10.2	122±13.5
after	133± 11.5	150 ± 11.3	110 ± 8.5*∗*	123 ± 10.1	118 ± 11.7

LDL, in mg/dL					
before	144±12.5	143 ± 12.4	121 ± 10.5	137 ± 11.7	131±12.1
after	139 ± 10.1*∗*	134 ± 11.2*∗*	104 ± 9.8*∗*	124 ± 10.3*∗*	129 ± 10.2

HDL, in mg/dL					
before	41.9±2.9	46.5 ± 3.7	49.8 ± 3.9	50.1 ± 4.2	44.0±2.2
after	42.2 ± 3.1	47.8 ± 3.9	50.0 ± 4.6	51.2 ± 4.4	45.1 ± 2.4

IOD, in *μ*M MDA					
before	142 ± 9.2	141 ± 12.7	115 ± 10.9	164 ± 5.8	177 ± 12.1
after	101 ± 8.7*∗∗*	92 ± 8.8*∗∗*	46 ± 4.5*∗∗*	42 ± 3.7*∗*	153 ± 11.9*∗*

LDL-Px, in ELISA × 10^3^					
before	310 ± 29	550 ± 61	664 ± 63	420 ± 45	450 ± 41
after	250 ± 24*∗*	350 ± 29*∗∗*	379 ± 34*∗∗*	130 ± 12*∗∗*	370 ± 32*∗*

Lipoprotein O_2_, in *μ*M					
before	4.07 ± 0.29	3.89 ± 0.35	3.86 ± 0.32	3.07 ± 0.29	3.67 ± 0.31
after	5.26 ± 0.33*∗*	4.64 ± 0.33*∗*	4.55 ± 0.39*∗*	3.44 ± 0.27	5.27 ± 0.39*∗*

StO_2_, in AUC mm					
before	81 ± 6.4	66 ± 5.2	67 ± 5.1	59 ± 4.4	76 ± 5.5
after	88 ± 6.9*∗*	79 ± 6.1*∗*	83 ± 7.1*∗*	79 ± 6.3*∗*	76 ± 6.3

*∗* p < 0.05, *∗∗*p < 0.001.

**Table 3:** Changes in sebum, and corneocyte parameters of the skin after supplementation with GA lycopene for one month.

Parameters before and after 4 weeks of trial	Groups with GA Lycopene supplementation				
	I	II	III	IV	V
Sebum droplet size, in *μ*m					
before	4.6 ± 1.11	3.96 ± 0.17	3.72 ± 0.43	3.89 ± 0.21	4.9 ± 0.53
after	5.1 ± 0.75*∗*	4.14 ± 0.11*∗*	4.20 ± 0.88*∗*	3.94 ± 0.22	4.9 ± 0.57

Corneocyte exfoliation rate^§^					
before	66 ± 6.8	82 ± 7.8	83 ± 9.3	87 ± 9.5	61 ± 6.2
after	63 ± 6.2	73 ± 12.0*∗*	76 ± 7.7*∗*	67 ± 13.5*∗*	60 ± 6.9

Corneocyte damage^§§^					
before	4.2 ± 0.98	7.19 ± 2.47	3.40 ± 0.97	3.50 ± 1.16	2.0 ± 1.98
after	2.5 ± 0.43*∗*	3.80 ± 1.23*∗∗*	2.41 ± 0.76*∗*	2.17 ± 0.52*∗*	1.8 ± 1.23

*∗* p < 0.05, *∗∗*p < 0.001.

^§^as an average number of single corneocytes in stratum cornea, ^§§^as an average number of cross-linked damaged corneocyte clusters in stratum cornea; each parameter was calculated in 40 randomly selected microscopic areas (x 1,000).

**Table 4:** Average relative species compositions of gut microbial communities for the intervention groups 7 mg GAL-MSFA, 30 mg GAL-MSFA, 30 GAL-PUFA at different doses at the beginning and at the end of the intervention study.

				7 mg	GAL MSFA	30 mg	.GAL MSFA	30 mg	GAL PUFA
Phyla	Family	Genera	Species	II_Day_0	II_Week_4	III_Day_0	III_Week_4	IV_Day_0	IV_Week_4
Bacteroidetes	Porphyromonadaceae	*Parabacteroides*	*distasonis*	0,36	0,09	0,04	0,04	0,04	0,05
Bacteroidetes	S24-7			0,73	1,72	0,15	0,48	0,07	0,2
Bacteroidetes	Porphyromonadaceae	*Parabacteroides*		0,33	0,1	0,68	0,09	0,05	0,24
Bacteroidetes	[Paraprevotellaceae]	*Paraprevotella*		0,11	0,05	0,26	0,03	0,01	0
Bacteroidetes	Bacteroidaceae	*Bacteroides*		8,59	2,58	0,91	3,14	0,74	0,95
Bacteroidetes	Bacteroidaceae	*Bacteroides*	*ovatus*	0,08	0,04	0,03	0,02	0,01	0
Bacteroidetes				0,77	1,14	0,31	0	0,08	0,02
Bacteroidetes	Prevotellaceae	*Prevotella*	*copri*	13,52	12,2	8,52	0,38	0,8	0,13
Bacteroidetes	Bacteroidaceae	*Bacteroides*	*caccae*	0,09	0,01	0,02	0,01	0	0
Bacteroidetes	Bacteroidaceae	*Bacteroides*	*Other*	0,15	0,04	0,04	0,1	0,09	0,26
Bacteroidetes	Prevotellaceae	*Prevotella*		0,26	0,19	0,38	0	0,2	0
Bacteroidetes	Prevotellaceae	*Prevotella*	*stercorea*	1,24	0,52	0,15	0	0,11	0,03
Bacteroidetes	Rikenellaceae			2,18	0,57	0,2	1,33	0,34	0,39
Bacteroidetes	Bacteroidaceae	*Bacteroides*	*plebeius*	0,49	0,99	0,15	1,22	2	0,01
Bacteroidetes	[Odoribacteraceae]	*Butyricimonas*		0,08	0,03	0,03	0,04	0,02	0,01
Bacteroidetes	[Paraprevotellaceae]	*[Prevotella]*		1,16	0,5	0,21	0	0,04	0,02
Bacteroidetes	[Barnesiellaceae]			0,71	0,07	0,02	0,1	0,05	0,06
Bacteroidetes	Bacteroidaceae	*Bacteroides*	*uniformis*	0,24	0,03	0,08	0,11	0,03	0,34

Actinobacteria	Bifidobacteriaceae	*Bifidobacterium*	*longum*	0,09	0,11	0,04	0,16	0,11	0,16
Actinobacteria	Bifidobacteriaceae	*Bifidobacterium*		0,15	0,54	0,03	0,5	0,73	2,31
Actinobacteria	Bifidobacteriaceae	*Bifidobacterium*	*adolescentis*	0,33	1,09	0,05	0,24	0,69	2,93
Actinobacteria	Actinomycetaceae	*Actinomyces*		0,02	0,01	0,04	0,35	0,04	0,02
Actinobacteria	Coriobacteriaceae	*Other*	*Other*	0	0	0,02	0,03	0,01	0,01
Actinobacteria	Coriobacteriaceae	*Eggerthella*	*lenta*	0,01	0	0,01	0,02	0,09	0,07
Actinobacteria	Coriobacteriaceae	*Atopobium*		0,01	0,01	0,01	0,09	0,02	0,02
Actinobacteria	Coriobacteriaceae	*Collinsella*	*aerofaciens*	0,96	0,58	0,59	1,2	1,57	0,94
Actinobacteria	Coriobacteriaceae			0,83	0,41	0,21	0,27	0,91	0,52
Actinobacteria	Coriobacteriaceae	*Adlercreutzia*		0,02	0,01	0,06	0,1	0,09	0,07
Actinobacteria	Coriobacteriaceae	*Slackia*		0,09	0,09	0,07	0,1	0,23	0,04

Firmicutes	Veillonellaceae	*Dialister*		0,3	0,82	1,54	0,09	1,2	7,23
Firmicutes	Lachnospiraceae	*Anaerostipes*		0,04	0,02	0,02	0,04	0,03	0,11
Firmicutes	Clostridiaceae	*Caloramator*		0,01	0	0,01	0,04	0,1	0
Firmicutes	Streptococcaceae	*Streptococcus*		0,13	0,21	0,8	8,14	0,19	2,26
Firmicutes	Clostridiaceae			0,95	0,75	0,82	4,8	5,63	0,94
Firmicutes	Lachnospiraceae	*Roseburia*		0,08	0,14	0,04	0,03	0,09	0,05
Firmicutes				6,99	6,64	16,32	10,2	8,45	5,98
Firmicutes	Clostridiaceae	*Other*	*Other*	0,03	0,01	0,03	0,22	0,53	0,04
Firmicutes	Lachnospiraceae	*[Ruminococcus]*	*Other*	0,01	0,01	0,02	0	0	0,01
Firmicutes	Lachnospiraceae	*[Ruminococcus]*	*gnavus*	0,04	0,04	0,05	0,06	0,13	0,06
Firmicutes	Lachnospiraceae	*Blautia*	*Other*	0,86	0,68	1,09	1,02	1,72	2,23
Firmicutes	Lachnospiraceae	*Blautia*		3,08	2,23	3,78	4,24	7,24	9,07
Firmicutes	Lachnospiraceae	*Coprococcus*	*Other*	0,13	0,22	0,36	0,15	0,15	0,15
Firmicutes	Ruminococcaceae	*Faecalibacterium*	*prausnitzii*	1,39	2,42	1,04	1	0,98	1,13
Firmicutes	Veillonellaceae	*Phascolarctobacterium*		0,39	0,17	0,04	0,02	3,69	0,01
Firmicutes	Ruminococcaceae			20,99	30,92	25,11	15,39	20,02	23,35
Firmicutes	Other	*Other*	*Other*	1,31	0,77	1,59	6,12	4,79	1,8
Firmicutes	Streptococcaceae	*Streptococcus*	*anginosus*	0,01	0	0,02	0,08	0	0,11
Firmicutes	Lachnospiraceae	*[Ruminococcus]*		0,2	0,31	0,17	0,19	0,26	0,17
Firmicutes	Erysipelotrichaceae			0,08	0,04	0,02	0,09	0,2	0,05
Firmicutes	Christensenellaceae			0,23	0,28	0,18	0,04	0,93	0,24
Firmicutes	Lachnospiraceae	*Shuttleworthia*		0,12	0,1	0,13	0,15	0,18	0,21
Firmicutes	Clostridiaceae	*Clostridium*		0,32	0,45	0,3	0,42	0,3	0,27
Firmicutes	Lachnospiraceae			7,01	8,06	8,82	7,27	9,5	8,18
Firmicutes	Ruminococcaceae	*Oscillospira*		1,71	1,44	1,69	2,56	1,98	1,66
Firmicutes	Erysipelotrichaceae	*[Eubacterium]*	*biforme*	0,53	0,27	0,38	1,03	0,78	0,09
Firmicutes	Lachnospiraceae	*Lachnospira*		0,25	0,11	0,23	0,04	0,23	0,21
Firmicutes	[Mogibacteriaceae]			0,17	0,11	0,06	0,08	0,29	0,11
Firmicutes	Lachnospiraceae	*Other*	*Other*	4,85	5,88	5,46	4,67	7,47	6,19
Firmicutes	Lachnospiraceae	*Coprococcus*		1,78	2,07	2,2	2,56	3,41	3,43
Firmicutes	Ruminococcaceae	*Ruminococcus*		1,85	2,11	4,7	2,11	1,49	4,73
Firmicutes	Lachnospiraceae	*Dorea*		1,88	2,8	1,77	2,15	2,76	2,13
Firmicutes	Ruminococcaceae	*Other*	*Other*	5,54	3,82	2,47	1,85	0,62	3,46
Firmicutes	Erysipelotrichaceae	*Catenibacterium*		0,63	0,38	2,25	1,18	2,4	2,88

Proteobacteria	Desulfovibrionaceae	*Bilophila*		0,02	0,01	0,01	0,01	0,11	0,02
Proteobacteria	Enterobacteriaceae			0,66	0,74	0,33	9,8	0,95	0,08
Proteobacteria	Alcaligenaceae	*Sutterella*		0,04	0,06	0,01	0	0,01	0

Verrucomicrobia	Verrucomicrobiaceae	*Akkermansia*	*muciniphila*	0,17	0,12	1,49	0,13	0,72	0,91

Tenericutes				0,19	0,12	0,2	0,08	0,01	0,21

TM7				0	0	0,01	0,01	0,02	0,01

Cyanobacteria				0,01	0,01	0,01	0,01	0,03	0,02

**Table 5:** Average relative species compositions of gut microbial communities for the intervention groups 7 mg DCL, 7 mg GAL-MSFA and DC at the beginning and at the end of the intervention study.

				7 mg_DCL	7 mg_DCL	7 mg_GAL-MSFA	7 mg_GAL-MSFA	DC	DC
				I_Day_0	I_Week_4	II_Day_0	II_Week_4	V_Day_0	V_Week_4
Bacteroidetes	Bacteroidaceae	*Bacteroides*	*Other*	1,08	0,21	0,15	0,04	0,12	0,04
Bacteroidetes	Bacteroidaceae	*Bacteroides*	*uniformis*	1,71	0,41	0,24	0,03	0,21	0,07
Bacteroidetes	Bacteroidaceae	*Bacteroides*		7,54	1,84	8,60	2,57	1,64	0,57
Bacteroidetes	Bacteroidaceae	*Bacteroides*	*eggerthii*	1,43	0,17	0,01	0,00	0,30	0,00
Bacteroidetes	Prevotellaceae	*Prevotella*	*copri*	2,05	22,30	13,55	12,19	0,28	2,72
Bacteroidetes	[Odoribacteraceae]	*Butyricimonas*		0,11	0,01	0,08	0,03	0,06	0,01
Bacteroidetes	Porphyromonadaceae	*Parabacteroides*	*distasonis*	0,46	0,07	0,36	0,09	0,13	0,05
Bacteroidetes	Porphyromonadaceae	Parabacteroides		0,48	0,05	0,33	0,10	0,26	0,32
TM7				0,02	0,00	0,01	0,00	0,00	0,01
Bacteroidetes	S24-7			0,00	0,00	0,72	1,71	1,55	1,56
Bacteroidetes	[Paraprevotellaceae]	*Paraprevotella*		0,58	0,05	0,11	0,05	0,04	0,00
Bacteroidetes	Bacteroidaceae	*Bacteroides*	*ovatus*	0,08	0,03	0,08	0,04	0,09	0,00
Bacteroidetes	Bacteroidaceae	*Bacteroides*	*caccae*	0,09	0,01	0,09	0,01	0,04	0,01
Bacteroidetes	[Barnesiellaceae]			0,22	0,06	0,70	0,07	0,03	0,01
Bacteroidetes	Prevotellaceae	*Prevotella*	*stercorea*	0,02	0,00	1,25	0,52	0,09	0,13
Bacteroidetes	Prevotellaceae	*Prevotella*		0,42	0,00	0,27	0,19	0,08	0,64
Bacteroidetes	Rikenellaceae			1,12	0,58	2,18	0,57	0,38	0,08

Actinobacteria	Coriobacteriaceae	*Collinsella*	*aerofaciens*	0,47	0,70	0,95	0,57	0,24	0,57
Actinobacteria	Coriobacteriaceae	*Slackia*		0,02	0,06	0,09	0,09	0,07	0,15
Actinobacteria	Bifidobacteriaceae	*Bifidobacterium*	*adolescentis*	0,06	0,62	0,33	1,09	1,13	0,58
Actinobacteria	Bifidobacteriaceae	*Bifidobacterium*	*longum*	0,37	0,83	0,09	0,11	0,23	0,08
Actinobacteria	Bifidobacteriaceae	*Bifidobacterium*		0,66	0,42	0,16	0,54	1,29	0,41
Actinobacteria	Coriobacteriaceae			0,20	0,52	0,83	0,41	1,36	1,50
Actinobacteria	Coriobacteriaceae	*Adlercreutzia*		0,03	0,03	0,02	0,01	0,03	0,05
Actinobacteria	Coriobacteriaceae	*Atopobium*		0,01	0,01	0,01	0,01	0,01	0,02
Actinobacteria	Actinomycetaceae	*Actinomyces*		0,03	0,04	0,02	0,01	0,02	0,04

Firmicutes	Ruminococcaceae	*Ruminococcus*		1,52	1,01	1,86	2,11	1,45	1,92
Firmicutes	Clostridiaceae	*Clostridium*		0,50	0,29	0,32	0,45	0,61	0,75
Firmicutes	Lachnospiraceae	*Shuttleworthia*		0,10	0,12	0,12	0,10	0,07	0,09
Firmicutes				7,10	7,06	6,98	6,65	5,43	5,16
Firmicutes	Lachnospiraceae	*Coprococcus*		2,19	2,06	1,78	2,06	1,66	1,76
Firmicutes	Ruminococcaceae	*Other*		0,83	1,10	5,54	3,82	0,87	0,54
Firmicutes	Clostridiaceae			1,88	0,99	0,95	0,75	0,58	0,94
Firmicutes	Lachnospiraceae	*Blautia*		1,22	1,61	0,87	0,68	1,37	1,49
Firmicutes	Christensenellaceae			0,51	0,52	0,23	0,27	0,36	1,61
Firmicutes	Other	*Other*		1,55	1,29	1,31	0,78	1,06	1,22
Firmicutes	Erysipelotrichaceae	*Catenibacterium*		0,10	0,79	0,63	0,38	0,83	1,11
Firmicutes	Lachnospiraceae	*Roseburia*		0,10	0,05	0,08	0,14	0,20	0,25
Firmicutes	Ruminococcaceae	*Ruminococcus*	*flavefaciens*	0,22	0,10	0,07	0,03	0,00	0,02
Firmicutes	Lachnospiraceae	*Blautia*		3,78	5,86	3,08	2,24	4,21	4,65
Firmicutes	Veillonellaceae	*Dialister*		1,08	1,00	0,30	0,82	3,64	1,25
Firmicutes	Ruminococcaceae	*Faecalibacterium*	*prausnitzii*	2,21	3,35	1,39	2,43	2,00	2,34
Firmicutes	Ruminococcaceae	*Oscillospira*		1,22	1,24	1,71	1,44	2,63	1,02
Firmicutes	Erysipelotrichaceae			0,06	0,06	0,08	0,04	0,06	0,02
Firmicutes	Streptococcaceae	S*treptococcus*		0,36	0,59	0,13	0,21	0,26	3,29
Firmicutes	Lactobacillaceae	*Lactobacillus*		0,02	0,01	0,00	0,02	0,02	0,14
Firmicutes	Ruminococcaceae			20,67	19,42	20,96	30,93	33,04	26,33
Firmicutes	Lachnospiraceae	*[Ruminococcus] *	*Other*	0,01	0,01	0,01	0,01	0,01	0,02
Firmicutes	[Mogibacteriaceae]			0,04	0,05	0,18	0,11	0,09	0,11
Firmicutes	Lachnospiraceae	*[Ruminococcus]*	*gnavus*	0,06	0,04	0,04	0,04	0,05	0,07
Firmicutes	Erysipelotrichaceae	*Bulleidia*	*p-1630-c5*	0,01	0,00	0,59	0,09	0,08	0,07
Firmicutes	Lachnospiraceae	*Anaerostipes*		0,04	0,04	0,04	0,02	0,05	0,09
Firmicutes	Lachnospiraceae	*Dorea*		3,64	2,31	1,87	2,81	1,61	3,00
Firmicutes	Veillonellaceae	*Veillonella*	*dispar*	0,04	0,05	0,01	0,03	0,16	0,27
Firmicutes	Veillonellaceae	*Phascolarctobacterium*		0,22	0,12	0,39	0,17	0,08	0,01
Firmicutes	Lachnospiraceae	*[Ruminococcus]*		0,27	0,17	0,20	0,30	0,24	0,41
Firmicutes	Clostridiaceae	*Other*	*Other*	0,06	0,03	0,03	0,01	0,01	0,01
Firmicutes	Lachnospiraceae	*Coprococcus*	*Other*	0,53	0,37	0,13	0,22	0,21	0,25
Firmicutes	Lachnospiraceae	*Lachnospira*		0,73	0,36	0,25	0,11	0,44	0,84
Firmicutes	Lachnospiraceae	*Other*	*Other*	7,50	5,93	4,84	5,90	6,08	10,35
Firmicutes	Erysipelotrichaceae	*[Eubacterium]*	*biforme*	0,14	0,13	0,53	0,27	0,09	0,32
Firmicutes	Lachnospiraceae			11,09	9,48	7,01	8,05	6,73	9,18

Proteobacteria	Enterobacteriaceae			0,35	0,08	0,67	0,74	2,51	0,65
Proteobacteria	Alcaligenaceae	*Sutterella*		0,07	0,04	0,04	0,06	0,03	0,19
Proteobacteria	Desulfovibrionaceae	*Bilophila*		0,01	0,01	0,02	0,01	0,02	0,01

Tenericutes				0,14	0,16	0,18	0,12	0,40	1,44

Verrucomicrobia	Verrucomicrobiaceae	*Akkermansia*	*muciniphila*	2,02	2,68	0,17	0,12	5,41	4,58

## Data Availability

The supporting results will be displayed on the publicly available website Lycotec.com. Moreover, the data that support the findings of this study are available from the corresponding author, Dr. Ivan M Petyaev, upon reasonable request.

## References

[1] Kardinaal A. F. M., van't Veer P., Kok F. (1993). Antioxidants in adipose tissue and risk of myocardial infarction: the EURAMIC study. *The Lancet*.

[2] Barber N. J., Barber J. (2002). Lycopene and prostate cancer. *Prostate Cancer and Prostatic Diseases*.

[3] Kim M. J., Kim H. (2015). Anticancer effect of lycopene in gastric carcinogenesis. *Journal of Cancer Prevention*.

[4] Zou Z.-Y., Xu X.-R., Lin X.-M. (2014). Effects of lutein and lycopene on carotid intima–media thickness in Chinese subjects with subclinical atherosclerosis: a randomised, double-blind, placebo-controlled trial. *British Journal of Nutrition*.

[5] Zigangirova N. A., Morgunova E. Y., Fedina E. D. (2017). Lycopene inhibits propagation of chlamydia infection. *Scientifica*.

[6] Schwarz S., Obermüller-Jevic U. C., Hellmis E., Koch W., Jacobi G., Biesalski H.-K. (2008). Lycopene inhibits disease progression in patients with benign prostate hyperplasia. *Journal of Nutrition*.

[7] Paur I., Lilleby W., Bøhn S. K. (2017). Tomato-based randomized controlled trial in prostate cancer patients: Effect on PSA. *Clinical Nutrition*.

[8] Stahl W., Heinrich U., Aust O., Tronnier H., Sies H. (2006). Lycopene-rich products and dietary photoprotection. *Photochemical & Photobiological Sciences*.

[9] Grether-Beck S., Marini A., Jaenicke T., Stahl W., Krutmann J. (2017). Molecular evidence that oral supplementation with lycopene or lutein protects human skin against ultraviolet radiation: results from a double-blinded, placebo-controlled, crossover study. *British Journal of Dermatology*.

[10] Ganji V., Kafai M. R. (2005). Population determinants of serum lycopene concentrations in the United States: Data from the Third National Health and Nutrition Examination Survey, 1988-1994. *Journal of Nutrition*.

[11] Müller L., Caris-Veyrat C., Lowe G., Böhm V. (2016). Lycopene and its antioxidant role in the prevention of cardiovascular diseases—a critical review. *Critical Reviews in Food Science and Nutrition*.

[12] Tzounis X., Rodriguez-Mateos A., Vulevic J., Gibson G. R., Kwik-Uribe C., Spencer J. P. E. (2011). Prebiotic evaluation of cocoa-derived flavanols in healthy humans by using a randomized, controlled, double-blind, crossover intervention study. *American Journal of Clinical Nutrition*.

[13] Petyaev I. M., Bashmakov Y. K. (2016). Cocobiota: implications for human health. *Journal of Nutrition and Metabolism*.

[14] Mursu J., Voutilainen S., Nurmi T. (2004). Dark chocolate consumption increases HDL cholesterol concentration and chocolate fatty acids may inhibit lipid peroxidation in healthy humans. *Free Radical Biology & Medicine*.

[15] Tokede O. A., Gaziano J. M., Djoussé L. (2011). Effects of cocoa products/dark chocolate on serum lipids: A meta-analysis. *European Journal of Clinical Nutrition*.

[16] Petyaev I. M., Dovgalevsky P. Y., Chalyk N. E., Klochkov V. A., Kyle N. H. (2016). Lycopene embedded into cocoa butter micelles of dark chocolate causes dose dependent decrease in serum lipids of hypercholesterolemic volunteers. *British Journal of Medicine Medical Research*.

[17] Petyaev I. M., Dovgalevsky P. Y., Klochkov V. A. (2018). Effect of lycopene supplementation on cardiovascular parameters and markers of inflammation and oxidation in patients with coronary vascular disease. *Food Science & Nutrition*.

[18] Gómez H., Mesquida J., Simon P. (2009). Characterization of tissue oxygen saturation and the vascular occlusion test: influence of measurement sites, probe sizes and deflation thresholds. *Circulation*.

[19] Chalyk N. E., Bandaletova T. Y., Kyle N. H., Petyaev I. M. (2017). Age-related differences in morphological characteristics of residual skin surface components collected from the surface of facial skin of healthy male volunteers. *Skin Research and Technology*.

[20] Øvreås L., Forney L., Daae F. L., Torsvik V. (1997). Distribution of bacterioplankton in meromictic lake Saelenvannet, as determined by denaturing gradient gel electrophoresis of PCR-amplified gene fragments coding for 16S rRNA. *Applied and Environmental Microbiology*.

[21] Krych Ł., Kot W., Bendtsen K. M. B., Hansen A. K., Vogensen F. K., Nielsen D. S. (2018). Have you tried spermine? A rapid and cost-effective method to eliminate dextran sodium sulfate inhibition of PCR and RT-PCR. *Journal of Microbiological Methods*.

[22] Edgar R. C. (2013). UPARSE: highly accurate OTU sequences from microbial amplicon reads. *Nature Methods*.

[23] McDonald D., Price M. N., Goodrich J. (2012). An improved Greengenes taxonomy with explicit ranks for ecological and evolutionary analyses of bacteria and archaea. *The ISME Journal*.

[24] Caporaso J. G., Kuczynski J., Stombaugh J. (2010). QIIME allows analysis of high-throughput community sequencing data. *Nature Methods*.

[25] Oksanen A. J., Blanchet F. G., Kindt R. vegan: Community Ecology Package. R Package.

[26] Diwadkar-Navsariwala V., Novotny J. A., Gustin D. M. (2003). A physiological pharmacokinetic model describing the disposition of lycopene in healthy men. *Journal of Lipid Research*.

[27] Yagi K. (1978). Lipid peroxide and human disease. *Chemistry and Physics of Lipids*.

[28] Petyaev I., Mitchinson M. M. J., Hunt J. V., Coussons P. J. (1998). Superoxide dismutase activity of antibodies purified from the human arteries andatherosclerotic lesions. *Biochemical Society Transactions*.

[29] Petyaev I. M., Vuylsteke A., Bethune D. W., Hunt J. V. (1998). Plasma oxygen during cardiopulmonary bypass: A comparison of blood oxygen levels with oxygen present in plasma lipid. *Clinical Science*.

[30] O'Neill C. A., Monteleone G., McLaughlin J. T., Paus R. (2016). The gut-skin axis in health and disease: A paradigm with therapeutic implications. *BioEssays*.

[31] Feng Q., Chen W., Wang Y. (2018). Gut microbiota: an integral moderator in health and disease. *Frontiers in Microbiology*.

[32] Marseglia L., Manti S., D’Angelo G. (2014). Oxidative stress in obesity: a critical component in human diseases. *International Journal of Molecular Sciences*.

[33] Costes F., Denis C., Roche F., Prieur F., Enjolras F., Barthélémy J.-C. (1999). Age-associated alteration of muscle oxygenation measured by near infrared spectroscopy during exercise. *Archives of Physiology and Biochemistry*.

[34] Petyaev I. M., Dovgalevsky P. Y., Klochkov V. A., Chalyk N. E., Kyle N. (2012). Clinical study: whey protein lycosome formulation improves vascular functions and plasma lipids with reduction of markers of inflammation and oxidative stress in prehypertension. *The Scientific World Journal*.

[35] Passi S., De Pità O., Puddu P., Littarru G. P. (2002). Lipophilic antioxidants in human sebum and aging. *Free Radical Research*.

[36] Macfarlane G., Cummings J. (1999). Probiotics and prebiotics: Can regulating the activities of intestinal bacteria benefit health?. *BMJ*.

[37] Gibson G. R., Hutkins R., Sanders M. E. (2017). Expert consensus document: the international scientific association for probiotics and prebiotics (ISAPP) consensus statement on the definition and scope of prebiotics. *Nature Reviews Gastroenterology & Hepatology*.

[38] Schnäbele K., Briviba K., Bub A., Roser S., Pool-Zobel B. L., Rechkemmer G. (2008). Effects of carrot and tomato juice consumption on faecal markers relevant to colon carcinogenesis in humans. *British Journal of Nutrition*.

[39] Głąbska D., Guzek D., Zakrzewska P., Włodarek D., Lech G. (2016). Lycopene, lutein and zeaxanthin may reduce faecal blood, mucus and pus but not abdominal pain in individuals with ulcerative colitis. *Nutrients*.

[40] Servin A. L. (2004). Antagonistic activities of lactobacilli and bifidobacteria against microbial pathogens. *FEMS Microbiology Reviews*.

[41] O'Callaghan A., van Sinderen D. (2016). Bifidobacteria and their role as members of the human gut microbiota. *Frontiers in Microbiology*.

[42] Arboleya S., Watkins C., Stanton C., Ross R. P. (2016). Gut Bifidobacteria Populations in Human Health and Aging. *Frontiers in Microbiology*.

[43] Bäckhed F., Ding H., Wang T. (2004). The gut microbiota as an environmental factor that regulates fat storage. *Proceedings of the National Acadamy of Sciences of the United States of America*.

[44] Turnbaugh P. J., Ley R. E., Mahowald M. A., Magrini V., Mardis E. R., Gordon J. I. (2006). An obesity-associated gut microbiome with increased capacity for energy harvest. *Nature*.

[45] Turnbaugh P. J., Hamady M., Yatsunenko T. (2009). A core gut microbiome in obese and lean twins. *Nature*.

[46] Liu R., Hong J., Xu X. (2017). Gut microbiome and serum metabolome alterations in obesity and after weight-loss intervention. *Nature Medicine*.

[47] Qin Y., Roberts J. D., Grimm S. A. (2018). An obesity-associated gut microbiome reprograms the intestinal epigenome and leads to altered colonic gene expression. *Genome Biology*.

